# Osmotically evoked PLCδ1-dependent translocation of ΔN-TRPV1 channels in rat supraoptic neurons

**DOI:** 10.1016/j.isci.2023.106258

**Published:** 2023-02-20

**Authors:** Kirk D. Haan, Sung Jin Park, Yoshikazu Nakamura, Kiyoko Fukami, Thomas E. Fisher

**Affiliations:** 1 Department of Anatomy, Physiology, and Pharmacology, College of Medicine, University of Saskatchewan, Saskatoon, SK, Canada; 2 Department of Applied Biological Science, Faculty of Science and Technology, Tokyo University of Science, Noda, Chiba, Japan; 3 Laboratory of Genome and Biosignals, School of Life Sciences, Tokyo University of Pharmacy and Life Sciences, Tokyo, Japan

**Keywords:** Cellular neuroscience, Cell biology

## Abstract

Osmoregulation is an essential homeostatic process that requires constant release of vasopressin during sustained increases in plasma osmolality. The magnocellular neurosecretory cells (MNCs) respond to increases in external osmolality through increases in the activity of ΔN-TRPV1 channels, which leads to increased action potential firing and vasopressin release. We show that sustained exposure of acutely isolated rat and mouse MNCs to hypertonic solutions (90 min) causes a reversible translocation of ΔN-TRPV1 channels from internal stores to the plasma membrane that depends on the activation of phospholipase C and on SNARE-dependent exocytosis. ΔN-TRPV1 channel translocation is absent in MNCs isolated from transgenic mice lacking the PLCδ1 isoform, suggesting that PLCδ1 is essential for triggering this process. The translocation of ΔN-TRPV1 channels to the cell surface could contribute to the maintenance of MNC excitability during sustained increases in osmolality. Our data therefore have important implications for the mechanisms underlying mammalian osmoregulation.

## Highlights

• Osmotic stimuli cause ΔN-TRPV1 channels to move to the surface of vasopressin neurons
• This process is reversible and depends on exocytosis and activation of PLC and PKC
• ΔN-TRPV1 translocation occurs in the VP neurons of water-deprived rats and mice
• ΔN-TRPV1 translocation is absent in the VP neurons of mice that do not express PLCδ1

## Graphical abstract

**Figure:**
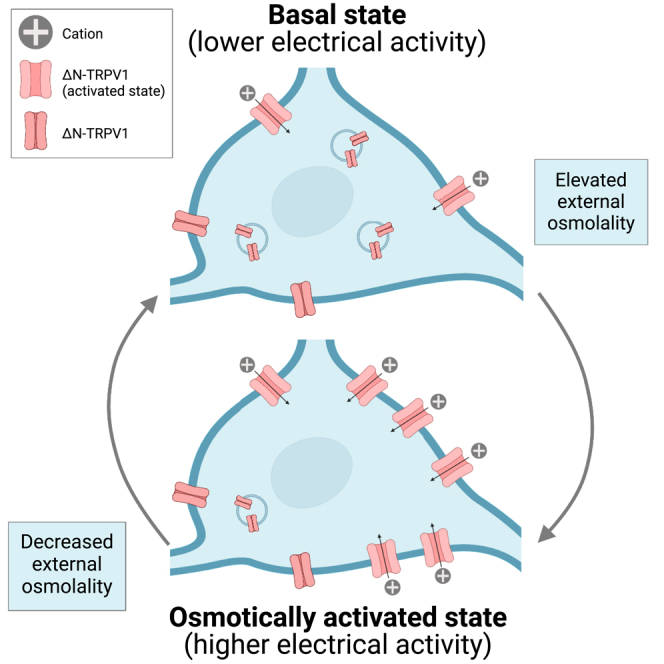

## Introduction

Osmoregulation is the essential homeostatic process that maintains the osmolality of our extracellular fluids within narrow limits despite constant changes in the intake and loss of salt and water.^1^^,^^2^^,^^3^ The primary hormone regulator of osmolality is vasopressin (VP), which acts on the kidney tubules to regulate urine production. When plasma osmolality is high, the plasma concentration of VP is high, which decreases the volume of urine production and thereby prevents water loss, whereas when plasma osmolality is low, the VP level is low, which enables water excretion. VP is released from the magnocellular neurosecretory cells (MNCs) of the hypothalamus, which are intrinsically osmosensitive^4^^,^^5^ and which receive excitatory inputs from other osmosensitive neurons.^1^ MNC osmosensitivity depends on the mechanical activation of an N-terminal variant of the transient receptor potential vanilloid1 cation channel (ΔN-TRPV1),^6^^,^^7^ leading to membrane depolarization, an increase in the frequency of action potentials, and VP release. These channels interact with the unique cytoskeleton of the MNCs^8^ and are activated by osmotically induced cell shrinkage through a process that involves both actin and tubulin.^9^^,^^10^ MNCs do not have the acute volume regulatory mechanisms found in many other neurons,^11^ and therefore the cells remain shrunk for at least several minutes when exposed to a hypertonic solution,^11^^,^^12^ resulting in sustained MNC firing and VP release.

More prolonged increases in osmolality (i.e., hours to days) have been shown to cause dramatic structural and functional changes in MNCs, including retraction of glial processes from around MNC somata and axon terminals,^13^^,^^14^ an increase in the density of subcortical and cytoplasmic actin networks,^15^ increases in the expression of many proteins,^16^ an increased density of a variety of channels and receptors on the MNC cell surface,^9^^,^^17^^,^^18^ and a marked hypertrophy of MNC somata.^13^^,^^14^ Osmotically induced hypertrophy occurs in acutely isolated MNC rat somata following tens of minutes of exposure to increased osmolality and requires the fusion of intracellular vesicles with the MNC plasma membrane.^12^

The expansion of the MNC plasma membrane during the hypertrophic response might diminish or eliminate the force exerted on ΔN-TRPV1 channels by osmotically induced cell shrinkage, which suggests that other processes might be involved in maintaining ΔN-TRPV1 activity and MNC excitability during sustained increases in osmolality. In other neurons, the translocation of TRPV1 channels to the neuronal cell surface can contribute to long-lasting increases in excitability,^19^^,^^20^^,^^21^^,^^22^^,^^23^ and such translocation can be triggered by the activation of phospholipase C (PLC) and/or protein kinase C (PKC).^22^^,^^24^ PLC is activated in isolated MNCs by exposure to hypertonic solution,^12^^,^^25^ and this activation is required for both osmotically induced MNC hypertrophy^12^ and for full activation of ΔN-TRPV1 currents.^25^ Transgenic mice lacking the PLCδ1 isoform^26^ display defective osmoregulation, and MNCs isolated from these mice show a diminished osmotic activation of ΔN-TRPV1 currents.^27^

These data led us to hypothesize that trafficking of ΔN-TRPV1 channels might be part of the MNC adaptation to sustained increases in external osmolality. We report here that exposure of isolated MNCs to hypertonic solution for 90 min, or depriving rats or mice of water for 24 h, causes a reversible translocation of ΔN-TRPV1 channels to the MNC plasma membrane. PLC activation is necessary for initiating and for maintaining channel translocation. Transgenic mice that do not express PLCδ1 do not undergo osmotically induced TRPV1 translocation, suggesting that this isoform is essential for activating this response. These observations have important implications for the ability of MNCs to maintain high levels of VP release during sustained increases in osmolality and therefore for our understanding of mammalian osmoregulation.

## Results

We showed previously that exposure of isolated rat MNCs to hypertonic solution for 90 min causes reversible somatic hypertrophy that depends on the activation of PLC and exocytotic fusion.^12^ We therefore performed experiments to determine whether translocation of ΔN-TRPV1 channels occurs in parallel. We used an antibody directed against an external epitope of the TRPV1 channels on live isolated MNCs to focus on channels in the MNC plasma membrane and to enable visualization of channel internalization during recovery.

### Sustained increases in osmolality cause reversible translocation of ΔN-TRPV1 channels to the plasma membrane of rat MNCs

**Figure 1:**
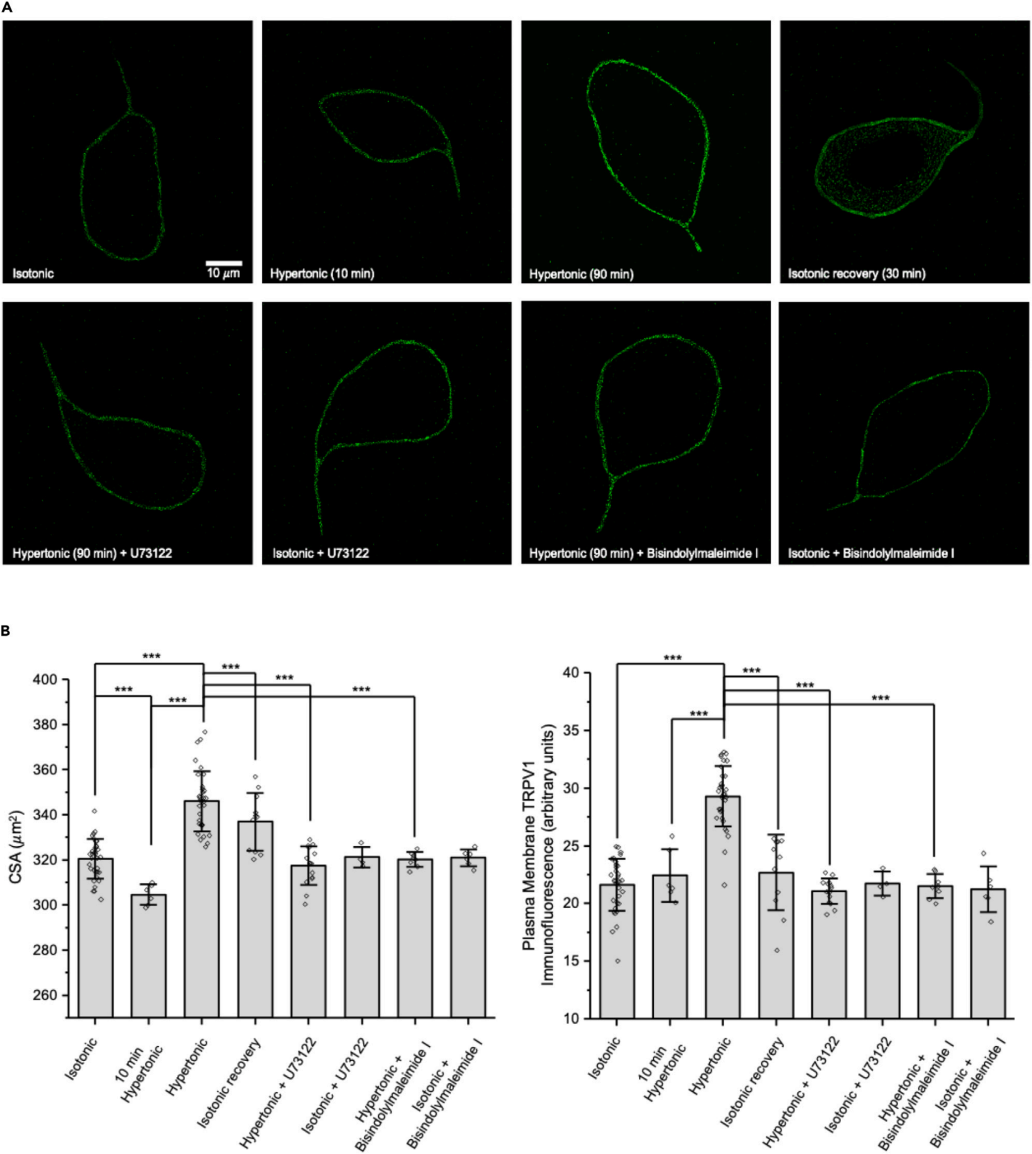
Osmotic stimulation causes a reversible translocation of ΔN-TRPV1 channels to the plasma membrane of isolated rat MNCs that depends on both PLC and PKC (A) Representative images of MNCs showing TRPV1 immunofluorescence in the listed conditions. The scale bar indicates 10 μm. (B) Bar-scatter plots of the cross-sectional area (CSA; μm^2^; left) and the plasma membrane TRPV1 labeling (in arbitrary units; a.u.: right) in each of the listed conditions. Data are expressed as means ± SD. The level of significance is indicated using asterisks – ∗ indicates p < 0.05, ∗∗ indicate p < 0.01, ∗∗∗ indicate p < 0.001, and the absence of an asterisk indicates no significant difference.

The upper panels in Figure 1A show representative images of TRPV1 immunofluorescence labeling of rat MNCs in isotonic solution, after treatment with hypertonic solution for 10 min, after treatment with hypertonic solution for 90 min, and after treatment with hypertonic solution for 90 min followed by return to isotonic solution for 30 min (“isotonic recovery”). Note that the cell exposed to hypertonic solution for 90 min appears larger and displays brighter TRPV1 immunofluorescence in its plasma membrane. Figure 1B has bar graphs displaying the effects of treatment on the cross-sectional area of MNCs (CSA, in μm^2^), and Figure 1C shows the effects of treatment on TRPV1 immunofluorescence labeling on the MNC plasma membrane in arbitrary units (a.u.). In the bar graphs depicting immunocytochemical data in this and all other figures, each symbol represents the mean of measured values from one dish of cells from a separate experiment, each of which included at least 8 cells, and the bars show the mean and standard deviation of those means. The data that was used to construct these bar graphs, and all other bar graphs in this paper, are summarized in Table S1. The CSA in isotonic solution was decreased significantly by exposure to hypertonic solution for 10 min but was significantly increased by exposure to hypertonic solution for 90 min. TRPV1 labeling in isotonic solution was not altered by exposure to hypertonic solution for 10 min but was increased significantly by exposure to hypertonic solution for 90 min. MNCs treated with hypertonic solution for 90 min and then returned to isotonic solution for 30 min (which is referred to as “isotonic recovery”) showed a partial recovery of the CSA and a nearly complete recovery of TRPV1 labeling intensity. These data confirm that treatment with hypertonic solution for 90 min causes a reversible MNC hypertrophy (as has been reported; Shah et al.^12^) and reveal a reversible increase in ΔN-TRPV1 channels in the MNC plasma membrane. Note that in all experiments described below, hypertonic treatments were applied for 90 min, unless otherwise noted.

### Osmotically induced ΔN-TRPV1 translocation in rat MNCs is prevented by inhibition of PLC or PKC

The lower panels in Figure 1A show representative images of TRPV1 immunofluorescence labeling of rat MNCs exposed to hypertonic solution in the presence of the PLC inhibitor U73122 (1 μM), MNCs exposed to U73122 in isotonic solution, MNCs exposed to hypertonic solution in the presence of the PKC inhibitor bisindolylmaleimide I (1 μM), and MNCs exposed to bisindolylmaleimide I in isotonic solution. Note that these MNCs appear to have CSAs and TRPV1 immunofluorescence in their plasma membrane similar to those of the MNC in isotonic solution in the upper right panel and lower than those seen in the MNC treated with hypertonic solution for 90 min. The data describing the effects of these drugs on CSA and TRPV1 immunofluorescence are summarized in Figures 1B and 1C, respectively. The CSAs of MNCs exposed to hypertonic solution in the presence of U73122 or bisindolylmaleimide I were not different than those of MNCs in isotonic solution and were significantly lower than those of MNCs exposed to hypertonic solutions with no inhibitors. The TRPV1 immunofluorescence in the plasma membrane of MNCs exposed to hypertonic solution in the presence of U73122 or bisindolylmaleimide I was not different than that of MNCs in isotonic solution and was significantly lower than that of MNCs exposed to hypertonic solutions with no inhibitors. Treatment with U73122 or bisindolylmaleimide I had no effect on either CSA or TRPV1 immunofluorescence when applied to MNCs in isotonic solution. We showed previously that U73122 and bisindolylmaleimide I prevent osmotically induced MNC hypertrophy^12^; these data demonstrate that they also prevent the osmotically induced increase in TRPV1 immunofluorescence in the MNC plasma membrane. Application of U77343 (1 μM), an inactive analogue of U73122, did not prevent the osmotically induced MNC hypertrophy or the increase in TRPV1 immunofluorescence. The mean values in MNCs treated with hypertonic saline in the presence of U77343 were not significantly different than those of MNCs treated with hypertonic saline alone (n = 6; data not shown).

### Recovery from osmotically induced ΔN-TRPV1 translocation in rat MNCs is prevented by the dynamin inhibitor dynasore

**Figure 2:**
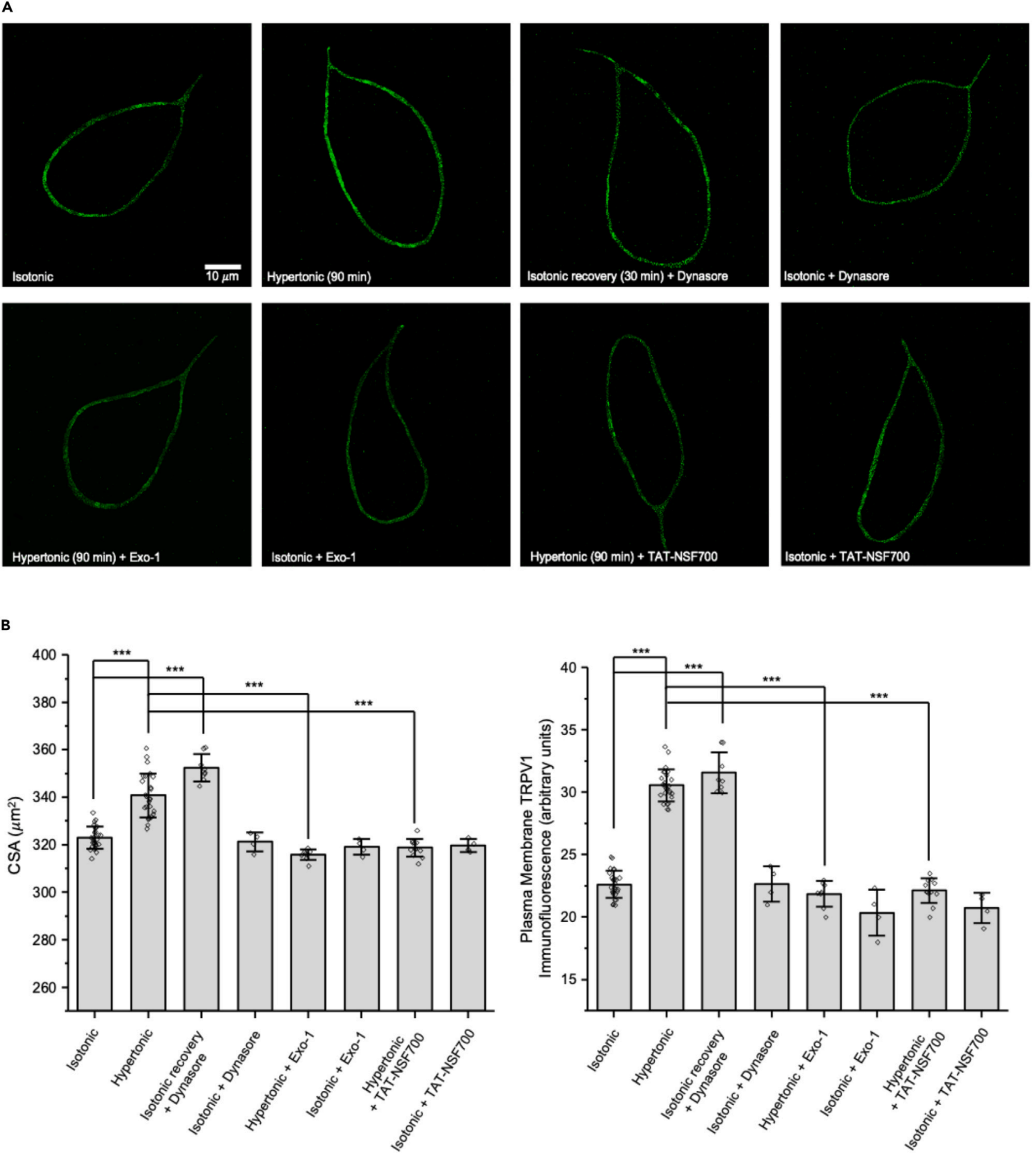
Recovery from osmotically induced ΔN-TRPV1 translocation in rat MNCs depends on dynamin-dependent endocytosis, and ΔN-TRPV1 translocation is prevented by blockers of exocytosis (A) Representative images of MNCs showing TRPV1 immunofluorescence in the listed conditions. The scale bar indicates 10 μm. (B) Bar-scatter plots of the cross-sectional area (CSA; μm^2^; left) and the plasma membrane TRPV1 labeling (in arbitrary units; a.u.: right) in each of the listed conditions. Data are expressed as means ± SD. The level of significance is indicated using asterisks – ∗ indicates p < 0.05, ∗∗ indicate p < 0.01, ∗∗∗ indicate p < 0.001, and the absence of an asterisk indicates no significant difference.

The upper panels in Figure 2A show representative images of TRPV1 immunofluorescence labeling in rat MNCs in isotonic solution, after treatment with hypertonic solution, after treatment with hypertonic solution followed by isotonic recovery in the presence of dynasore (80 μM; an inhibitor of dynamin), and in isotonic solution in the presence of dynasore. Note that unlike in Figure 1, treatment with isotonic solution following exposure to hypertonic solution did not appear to reverse the osmotically induced increase in TRPV1 labeling when in the presence of dynamin. The data for CSA and TRPV1 immunofluorescence are summarized in the left and right graphs of Figure 2B. The CSA and the TRPV1 immunofluorescence labeling of the MNC plasma membrane in isotonic solution were increased by treatment with hypertonic solution, but there was no reversal of the increase in CSA or the increase in TRPV1 labeling following exposure to isotonic solution in the presence of dynasore. Dynasore had no effect on either parameter when applied to MNCs in isotonic solution. These data indicate that dynasore inhibits the recovery from the osmotically induced increases in CSA and TRPV1 labeling shown in Figure 1.

### Osmotically induced ΔN-TRPV1 translocation in rat MNCs depends on Golgi membrane trafficking and on SNARE-mediated exocytosis

The lower panels of Figure 2A show representative images of TRPV1 immunofluorescence in rat MNCs exposed to hypertonic solution in the presence of Exo-1 (80 μM), treated with Exo-1 in isotonic solution, exposed to hypertonic solution in the presence of TAT-NSF700 (1.2 μM), and treated with TAT-NSF700 in isotonic solution. Exo-1 inhibits exocytotic fusion by inducing collapse of the Golgi into the ER,^28^ and TAT-NSF700 is a cell-permeant peptide that interferes with the function of *N*-ethylmaleimide-sensitive factor (NSF), which is a key component of the soluble NSF attachment proteins receptor (SNARE) complex that mediates exocytotic membrane fusion.^12^^,^^29^ The data for CSA and TRPV1 labeling are summarized in the left and right graphs of Figure 2B. The osmotically induced increase in CSA was prevented by the presence of Exo-1 and by the presence of TAT-NSF700. Similarly, the osmotically induced increase in TRPV1 immunofluorescence labeling in the MNC plasma membrane was prevented by the presence of Exo-1 and by the presence of TAT-NSF700. Treatment with Exo-1 or TAT-NSF700 had no effect on either CSA or TRPV1 immunofluorescence when applied to MNCs in isotonic solution. These data indicate that the osmotically induced increases in CSA and TRPV1 immunofluorescence in the plasma membrane both depend on exocytotic fusion of internal membranes with the MNC plasma membrane.

### Osmotically induced ΔN-TRPV1 translocation in rat MNCs elicits an increase in ΔN-TRPV1-mediated cation currents

**Figure 3:**
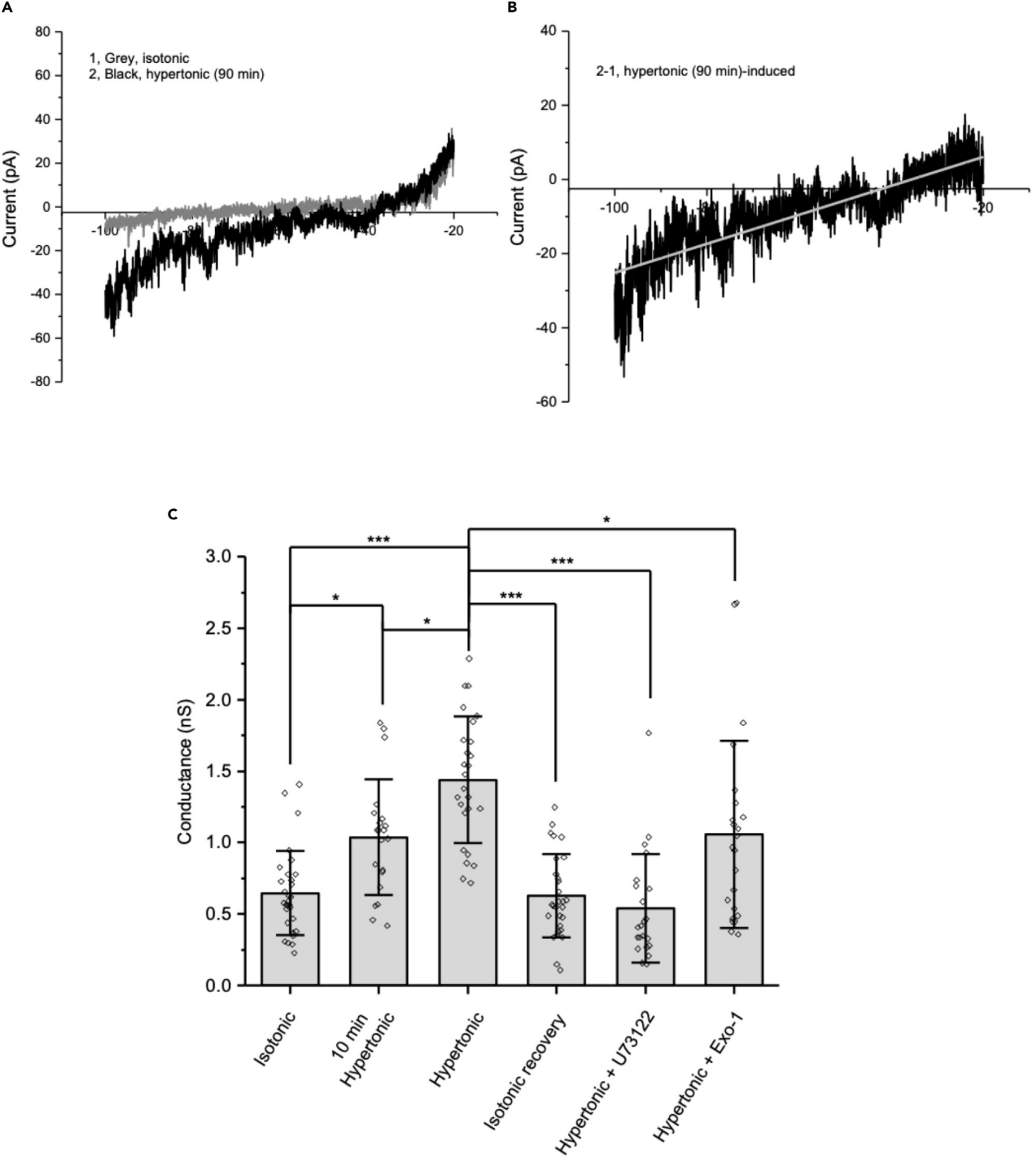
The osmotically induced increase in ΔN-TRPV1 current in rat MNCs was enhanced by a 90-min exposure to hypertonic solution, was reversible, and was blocked by inhibition of PLC or exocytotic fusion (A) Representative *I-V* relationships plotted from current traces in isotonic for 30 min (gray) and hypertonic for 90 min (black). (B) *I-V* relationship showing the difference between the two current traces shown in A. (C) Bar-scatter plot illustrating the amplitude of the current (expressed as slope conductance in nS) evoked under the listed conditions. Data are expressed as means ± SD. The level of significance is indicated using asterisks – ∗ indicates p < 0.05, ∗∗ indicate p < 0.01, ∗∗∗ indicate p < 0.001, and the absence of an asterisk indicates no significant difference.

The activation of ΔN-TRPV1 channels is a critical component of the osmotic activation of rat and mouse MNCs.^1^^,^^7^^,^^27^ Figure 3A shows currents measured in rat MNCs using a ramp protocol under whole-cell patch clamp in isotonic solution (gray line) and following treatment in hypertonic solution for 90 min (black line). Figure 3B shows the difference between the two traces (i.e., the osmotically induced current). The mean reversal potential of the osmotically induced currents was −40.5 ± 7.3 mV (n = 17), which is consistent with that observed previously in rat MNCs^5^^,^^25^ and in mouse MNCs^30^ for ΔN-TRPV1-mediated currents. We showed previously that this current is blocked by the TRPV1-selective blocker SB366791.^27^ The results of these experiments are summarized in Figure 3C. The mean slope conductance measured in MNCs at −60 mV in isotonic solution was significantly greater in MNCs treated with hypertonic solution for 10 min and was significantly higher than that in MNCs treated with hypertonic solution for 90 min. These data are consistent with a rapidly induced increase in ΔN-TRPV1 currents, as has been shown to occur through the mechanosensitivity of ΔN-TRPV1 channels,^5^^,^^25^ and suggest a second, slower phase of increase in ΔN-TRPV1 current that is evident after 90 min of exposure to hypertonic solution. MNCs treated with hypertonic solution for 90 min followed by isotonic solution for 30 min showed a reduction of slope conductance compared to those treated only with hypertonic solution, suggesting that both phases of osmotically induced increases in ΔN-TRPV1 currents are rapidly reversible. The increase in slope conductance of current induced by treatment with hypertonic solution for 90 min was blocked by U73122, suggesting that the increase in current depends on the activation of PLC. The increase in slope conductance of current induced by treatment with hypertonic solution was also significantly decreased by Exo-1, suggesting that the increase in current depends on exocytotic fusion with the MNC plasma membrane. These data support the hypothesis that the slower increase in current that occurs during treatment with hypertrophic solution depends in part on ΔN-TRPV1 translocation from internal stores to the MNC plasma membrane.

### MNCs isolated from PLCδ1 KO mice do not exhibit osmotically induced ΔN-TRPV1 translocation

**Figure 4:**
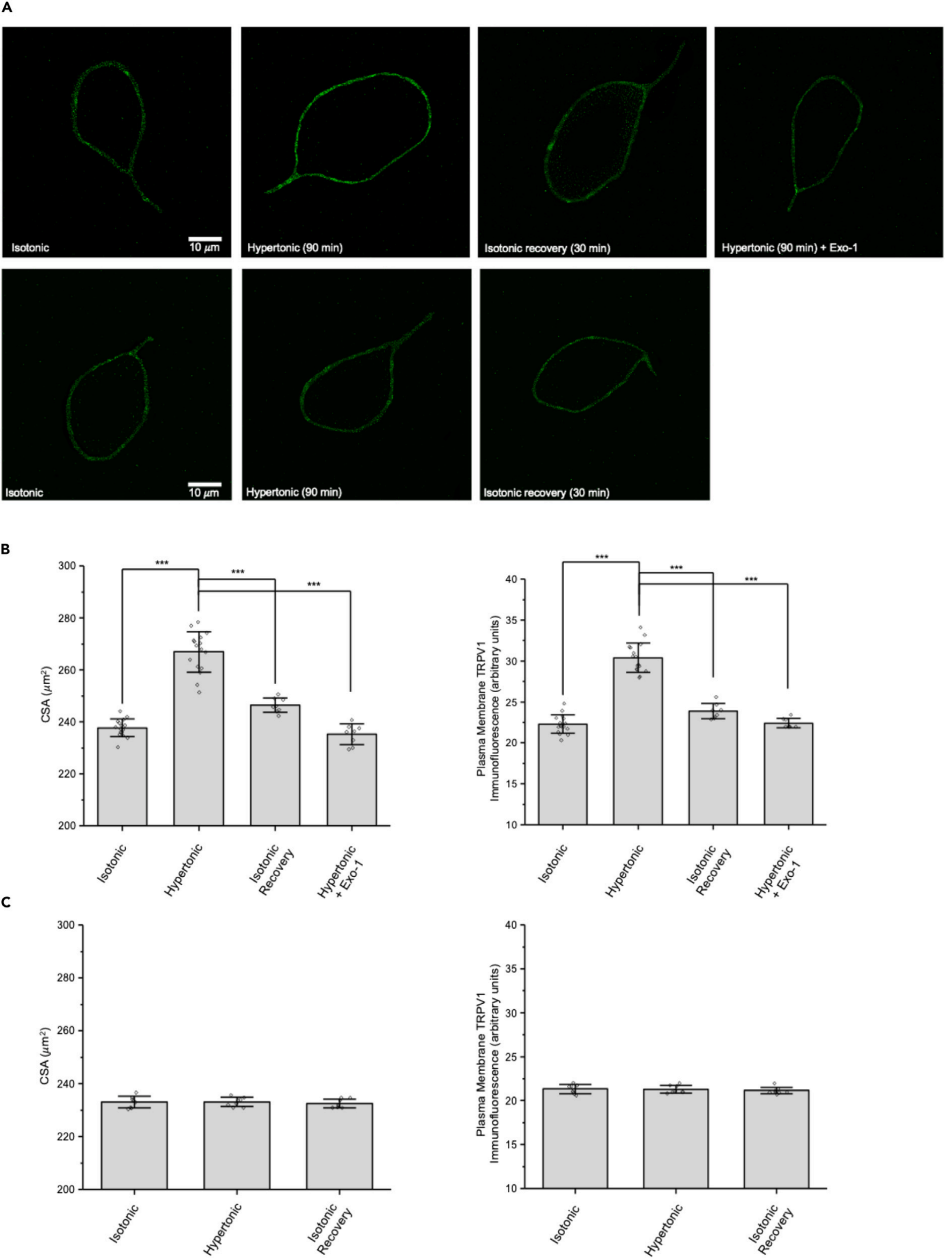
Osmotic stimulation causes a reversible translocation of ΔN-TRPV1 channels to the plasma membrane of MNCs isolated from control mice but not PLCδ1 knockout mice (A) The upper panels show representative images of MNCs isolated from control mice showing TRPV1 immunofluorescence in the listed conditions. The lower panels show representative images of MNCs isolated from PLCδ1 knockout mice showing TRPV1 immunofluorescence in the listed conditions. The scale bar indicates 10 μm. (B) Bar-scatter plots of the cross-sectional area (CSA; μm^2^; left) and the plasma membrane TRPV1 labeling (in arbitrary units; a.u.: right) for MNCs isolated from control mice in each of the conditions shown in the upper panels. (C) Bar-scatter plots of the cross-sectional area (CSA; μm^2^; left) and the plasma membrane TRPV1 labeling (in arbitrary units; a.u.: right) for MNCs isolated from PLCδ1 knockout mice in each of the conditions shown in the lower panels. Data are expressed as means ± SD. The level of significance is indicated using asterisks – ∗ indicates p < 0.05, ∗∗ indicate p < 0.01, ∗∗∗ indicate p < 0.001, and the absence of an asterisk indicates no significant difference.

Osmotically induced activation of ΔN-TRPV1 current is impaired in PLCδ1 knockout (KO) mice, as is osmoregulation.^27^ We therefore sought to test whether osmotically induced ΔN-TRPV1 translocation occurs in MNCs isolated from control (C57BL/6J) and PLCδ1 KO mice. The upper panels in Figure 4A show representative images of TRPV1 immunofluorescence labeling in MNCs isolated from control mice in isotonic solution, following treatment with hypertonic solution, following treatment with hypertonic solution followed by isotonic recovery, and following treatment with hypertonic solution in the presence of Exo-1. The data for CSA and TRPV1 immunofluorescence labeling are summarized in the left and right graphs in Figure 4B. The mean CSA of mouse MNCs in isotonic solution was increased significantly by exposure to hypertonic solution, and subsequent treatment with isotonic solution caused a nearly complete recovery of the CSA to the value in isotonic solution. The increase in CSA caused by hypertonic solution was prevented by the presence of Exo-1 (80 μM). Similarly, TRPV1 immunofluorescence on the MNC plasma membrane in isotonic solution was increased significantly by exposure to hypertonic solution, and subsequent treatment with isotonic solution caused a nearly complete recovery to the value observed in isotonic solution. The increase in TRPV1 immunofluorescence on the MNC plasma membrane caused by hypertonic solution was prevented by the presence of Exo-1. These data suggest that osmotically induced ΔN-TRPV1 translocation occurs in MNCs isolated from control mice as it does in rat MNCs.

The lower panels in Figure 4A show representative images of TRPV1 immunofluorescence in MNCs isolated from PLCδ1 KO mice following treatment with isotonic solution, hypertonic solution, and hypertonic solution followed by isotonic recovery. The data for CSA and TRPV1 immunofluorescence labeling are summarized in the left and right graphs in Figure 4C. Neither the CSA nor the TRPV1 immunofluorescence was different in any of the conditions. These data suggest that osmotically induced ΔN-TRPV1 translocation does not occur in MNCs isolated from PLCδ1 KO mice as it does in MNCs isolated from control mice or from rats.

### MNCs isolated from water-deprived rats and mice display reversible increases in plasma membrane ΔN-TRPV1 channel density and increases in channel activity

**Figure 5:**
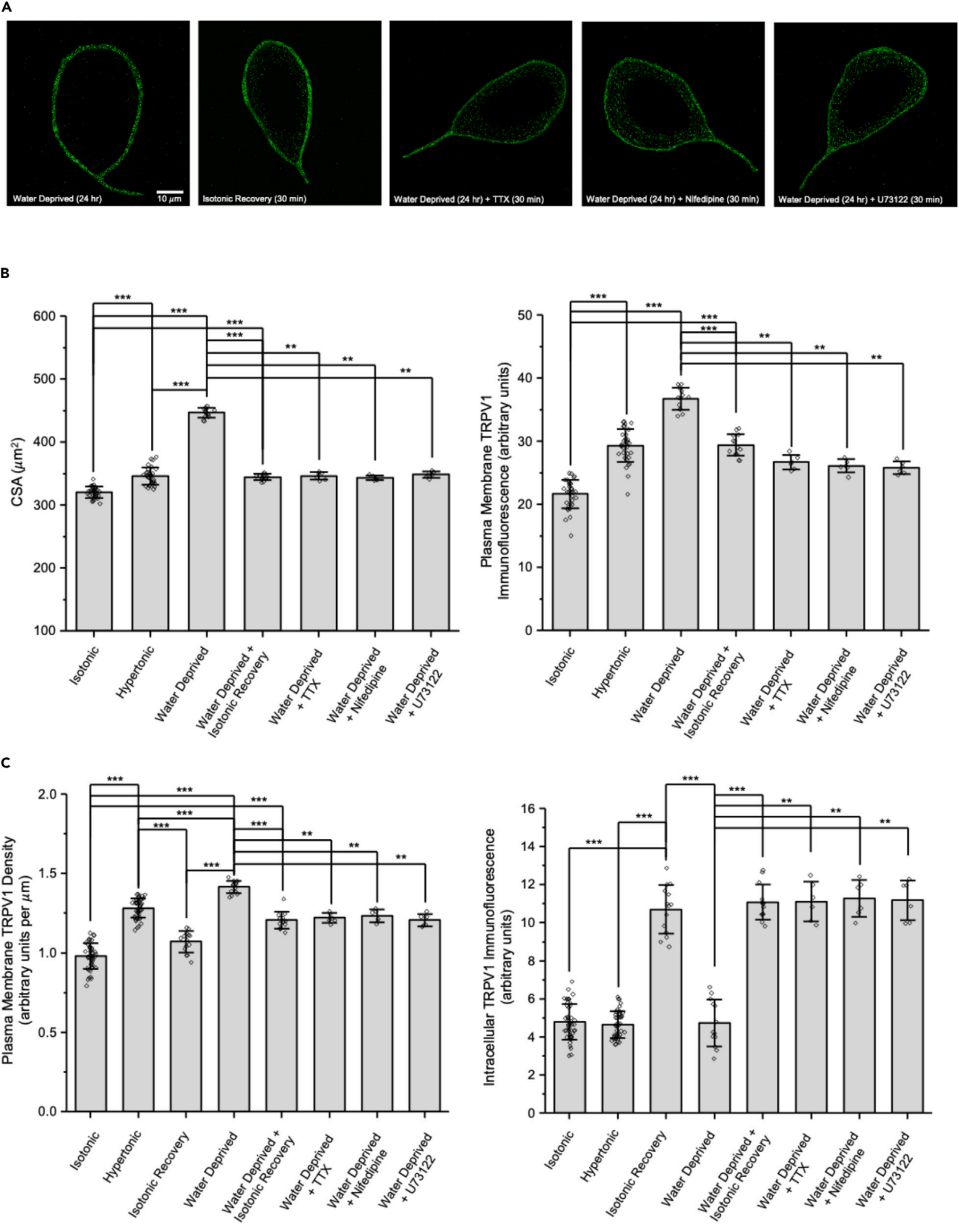
MNCs isolated from rats that were water-deprived for 24 h showed a large and reversible increase in CSA and ΔN-TRPV1 immunofluorescence (A) Representative images showing TRPV1 immunofluorescence in MNCs isolated from water-deprived rats in the listed conditions. (B) Bar-scatter plots showing the cross-sectional area (CSA; μm^2^; left) and TRPV1 immunofluorescence labeling in arbitrary units (a.u.) in the MNC plasma membrane (right) in the listed conditions. (C) Bar-scatter plots showing the changes in density of TRPV immunofluorescence on the MNC plasma membrane (a.u. per μm; left) and the change in internal TRPV1 immunofluorescence (a.u.; right) in the listed conditions. Data are expressed as means ± SD. The level of significance is indicated using asterisks – ∗ indicates p < 0.05, ∗∗ indicate p < 0.01, ∗∗∗ indicate p < 0.001, and the absence of an asterisk indicates no significant difference.

The panels in Figure 5A show representative images of TRPV1 immunofluorescence labeling in MNCs isolated from rats that had been deprived of water for 24 h and maintained in hypertonic solution during isolation, MNCs that underwent similar treatment but were then exposed to isotonic solution for 30 min, and MNCs that remained in hypertonic solution and were treated with the Na^+^ channel blocker tetrodotoxin (TTX), the L-type Ca^2+^ channel blocker nifedipine, or the PLC inhibitor U73122. Note that the MNC that remained in hypertonic solution with no treatment was large, had a high intensity of TRPV1 immunofluorescence on its plasma membrane, and showed little internal staining, whereas the representative MNCs for the other four conditions were markedly smaller, had lower intensity of TRPV1 immunofluorescence on their plasma membranes, and showed much greater internal staining.

The bar graph on the left of Figure 5B shows that the mean CSA of MNCs isolated from water-deprived rats was significantly larger than that of MNCs isolated from normally hydrated rats (data from Figure 1B) even those that were exposed to hypertonic solution (data from Figure 1B) and that the increase in CSA was reversed by returning the MNCs to isotonic solution or by treating with TTX, nifedipine, or U73122. The bar graph on the right of Figure 5B summarizes the data on TRPV1 immunofluorescence on the MNC plasma membrane. The TRPV1 immunofluorescence on the plasma membrane of the MNCs isolated from water-deprived rats maintained in hypertonic solution was significantly greater than that of MNCs isolated from normally hydrated rats (data from Figure 1C) even those that were exposed to hypertonic solution (data from Figure 1C). TRPV1 immunofluorescence was significantly lower in MNCs from water-deprived rats that were exposed to isotonic solution for 30 min or that were treated with TTX, nifedipine, or U73122. The bar graph on the left of Figure 5C summarizes the change in the density of TRPV1 immunofluorescence on the MNC plasma membrane (in arbitrary units per μm) in the listed conditions. The density of TRPV1 immunofluorescence in isotonic solution was significantly greater in MNCs exposed to hypertonic solution for 90 min and even greater in MNCs isolated from water-deprived rats. The increased density of TRPV1 immunofluorescence was decreased by a return to isotonic solution or by treatment with TTX, nifedipine, or U73122. The bar graph on right shows that intracellular TRPV1 immunofluorescence is low in MNCs isolated from normally hydrated rats in isotonic or hypertonic solution and in MNCs isolated from water-deprived rats in hypertonic solution. It is much higher, however, following treatment with isotonic solution of MNCs in hypertonic solution (isolated from either normally hydrated or water-deprived rats) and in MNCs from water-deprived rats that remained in hypertonic solution but were treated with any of the three blockers.

**Figure 6:**
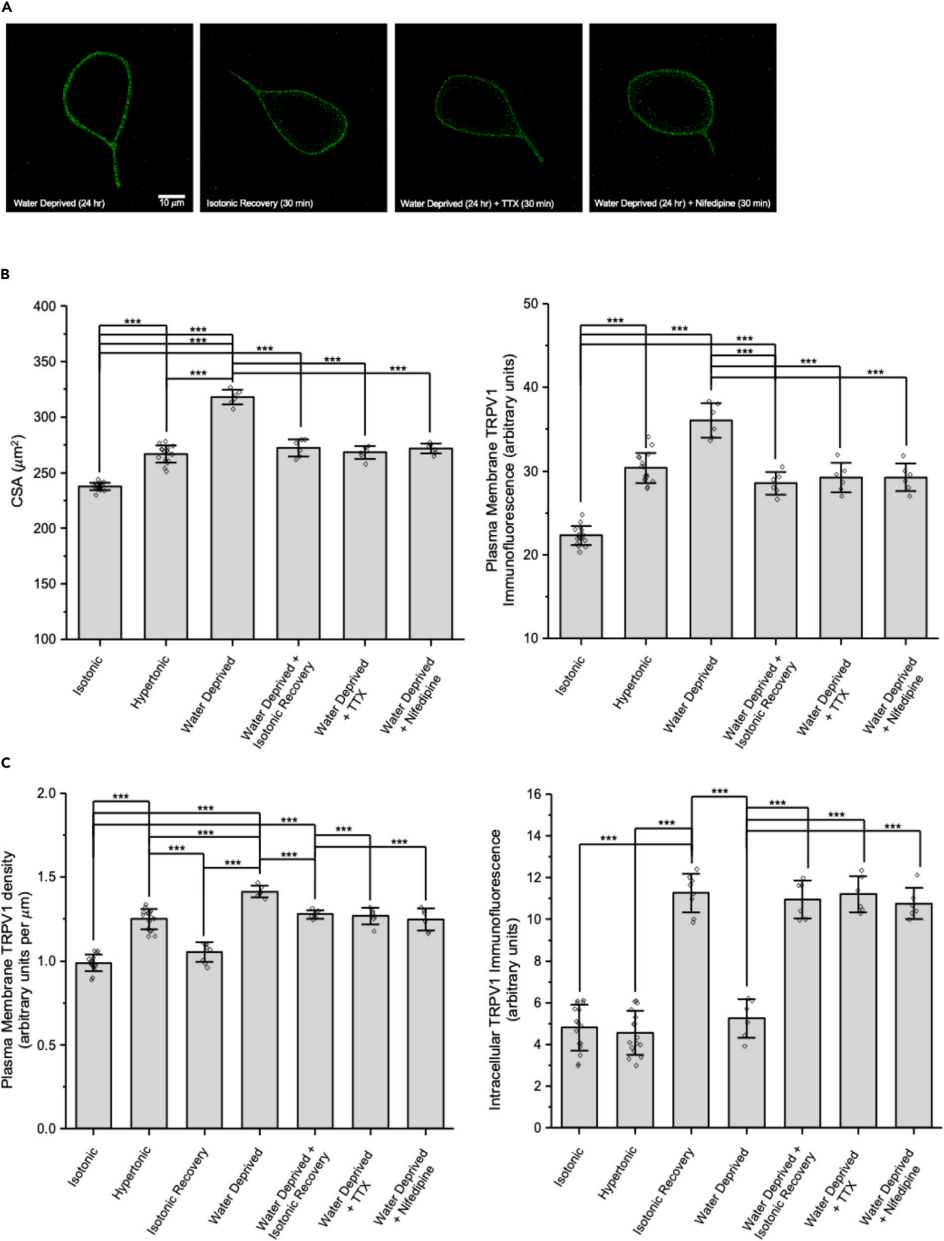
MNCs isolated from mice that were water-deprived for 24 h showed a large and reversible increase in CSA and ΔN-TRPV1 immunofluorescence (A) Representative images showing TRPV1 immunofluorescence in MNCs isolated from water-deprived rats in the listed conditions. (B) Bar-scatter plots showing the cross-sectional area (CSA; μm^2^; left) and TRPV1 immunofluorescence labeling in arbitrary units (a.u.) in the MNC plasma membrane (right) in the listed conditions. (C) Bar-scatter plots showing the changes in density of TRPV immunofluorescence on the MNC plasma membrane (a.u. per μm; left) and the change in internal TRPV1 immunofluorescence (a. u.; right) in the listed conditions. Data are expressed as means ± SD. The level of significance is indicated using asterisks – ∗ indicates p < 0.05, ∗∗ indicate p < 0.01, ∗∗∗ indicate p < 0.001, and the absence of an asterisk indicates no significant difference.

Figure 6 demonstrates that water deprivation of mice causes similar changes in MNCs. The panels in Figure 6A show representative images of TRPV1 immunofluorescence labeling in MNCs isolated from mice that had been deprived of water for 24 h and maintained in hypertonic solution during isolation, MNCs that underwent similar treatment but were then exposed to isotonic solution for 30 min, and MNCs that remained in hypertonic solution and were treated with TTX or nifedipine. The bar graphs in Figure 6B display the CSA and plasma membrane TRPV1 immunofluorescence of MNCs in each condition. (The data for MNCs isolated from normally hydrated mice in isotonic and hypertonic solution were obtained from Figure 4B). Water deprivation caused a large increase in both CSA and TRPV1 immunofluorescence, and exposure to isotonic solution, TTX, or nifedipine caused a decrease in both values. The bar graph on the left of Figure 6C summarizes the change in the density of TRPV1 immunofluorescence on the MNC plasma membrane (in arbitrary units per μm) in the listed conditions. The density of TRPV1 immunofluorescence in isotonic solution was significantly greater in MNCs exposed to hypertonic solution for 90 min and even greater in MNCs isolated from water-deprived mice, compared to that of MNCs in isotonic solution. The increased density of TRPV1 immunofluorescence was decreased by a return to isotonic solution or by treatment with TTX or nifedipine. The bar graph on right shows that intracellular TRPV1 immunofluorescence is low in MNCs isolated from normally hydrated mice in isotonic or hypertonic solution and in MNCs isolated from water-deprived mice in hypertonic solution. It is much higher, however, following treatment with isotonic solution of MNCs in hypertonic solution (isolated from either normally hydrated or water-deprived mice) and in MNCs from water-deprived mice that remained in hypertonic solution but were treated with TTX or nifedipine.

These data suggest that water deprivation of rats and mice causes a larger hypertrophic response and a greater increase in TRPV1 channels on the MNC plasma membrane than does exposure of isolated MNCs to hypertonic solution for 90 min but that these responses are still reversible upon exposure to isotonic solution. The observation that the density of TRPV1 immunofluorescence increases upon hypertonic treatment indicates that the intracellular membranes responsible for TRPV1 translocation have a higher concentration of TRPV1 than does the MNC plasma membrane. Measurements of internal TRPV1 immunofluorescence suggest that the reversal of the increase of TRPV1 immunofluorescence on the MNC membrane induced by a return to a solution of normal osmolality is due to internalization of the channels. The data showing that treatment of the MNCs with TTX, nifedipine, or U73122 in the continued presence of hypertonic solution causes reductions in CSA and TRPV1 plasma membrane immunofluorescence, and an increase in intracellular staining, suggest that action potential firing, Ca^2+^ influx, and PLC activity are required to maintain the MNCs in the hypertrophied state and to maintain the TRPV1 channels in the plasma membrane.

**Figure 7:**
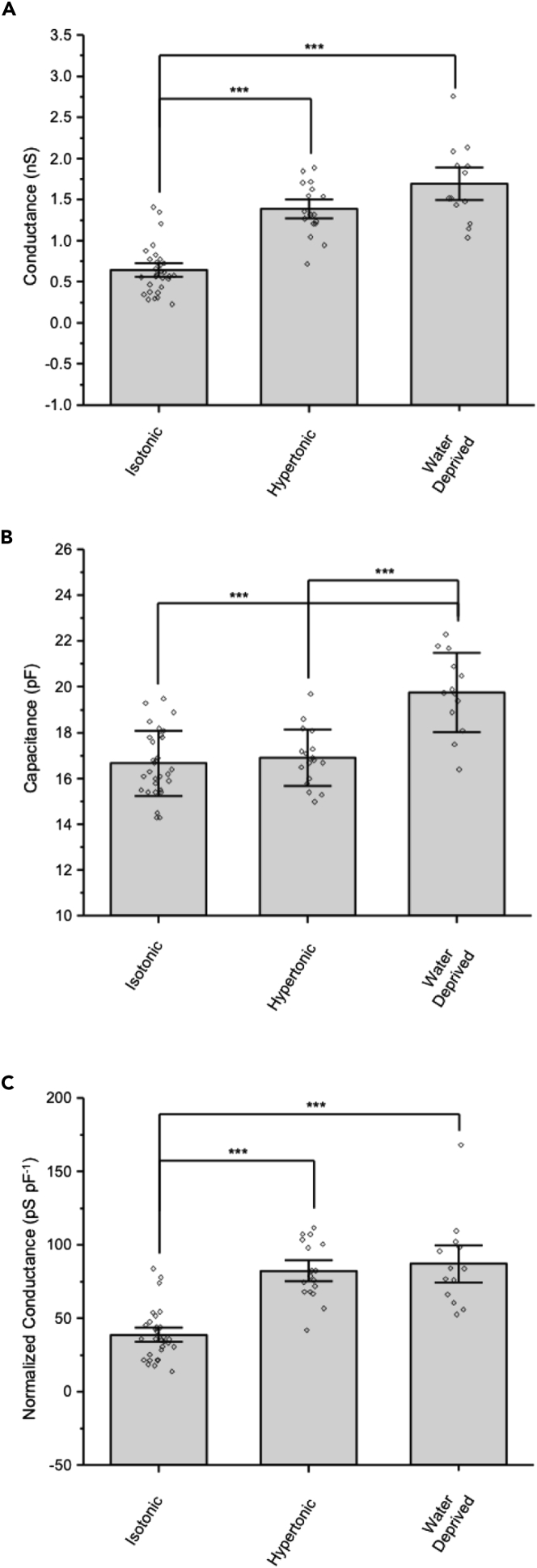
MNCs isolated from rats that were water-deprived for 24 h show a large increase in ΔN-TRPV1 currents relative to that caused by a 90 min incubation in hypertonic solution (A–C) Bar-scatter plots showing (A) the conductance (nS), (B) the capacitance (pF), and (C) the normalized conductance (pSpF^−1^) of MNCs isolated from normally hydrated rats in isotonic solution and following treatment with hypertonic solution for 90 min and of MNCs isolated from rats that had been deprived of water for 24 h. Each symbol in the bar-scatter plot represents a recording from one isolated MNC. Data are expressed as means ± SD. The level of significance is indicated using asterisks – ∗ indicates p < 0.05, ∗∗ indicate p < 0.01, ∗∗∗ indicate p < 0.001, and the absence of an asterisk indicates no significant difference.

The graphs in Figure 7 summarize data comparing the amplitude of currents in and the whole-cell capacitance of MNCs isolated from normally hydrated rats that were exposed to hypertonic treatment to those of MNCs isolated from water-deprived rats and maintained in hypertonic solutions. Figure 7A shows that the mean slope conductance in MNCs in isotonic solution was significantly greater in MNCs exposed to hypertonic solution and in MNCs isolated from water-deprived rats. Figure 7B shows that the capacitance measured in MNCs in isotonic solution was not significantly different in MNCs exposed to hypertonic solution but was significantly higher in MNCs isolated from water-deprived rats compared to the capacitance measured in both isotonic solution and hypertonic solution. The increase in capacitance caused by water deprivation was similar to what we observed previously.^31^ We have previously reported a small (about 7%) but significant increase in capacitance in isolated cells exposed to hypertonic solution for 90 min,^12^ but that paper had a larger sample size than the result reported here. Figure 7C shows that the normalized conductance (pSpF^−1^) of MNCs in isotonic solution was significantly greater in MNCs exposed to hypertonic solution and in MNCs isolated from water-deprived rats. These data are consistent with the imaging data in suggesting that TRPV1 channels are translocated to the MNC plasma membrane following water deprivation and further suggest that these channels remain active in the hypertrophied MNCs.

## Discussion

We showed previously that exposure of acutely isolated rat MNCs to hypertonic solutions causes hypertrophy over tens of minutes and that this process depends on Ca^2+^ influx through L-type Ca^2+^ channels and the Ca^2+^-dependent activation of PLC.^12^ Osmotically evoked hypertrophy depends on exocytotic fusion of internal membranes with the MNC plasma membrane, and the recovery from hypertrophy is blocked by the dynamin inhibitor dynasore.^12^ (It has been shown, however, that dynasore can have dynamin-independent effects on membrane function,^32^ and it is possible that the inhibition of endocytosis that we observed depends on a mechanism other than dynamin inhibition.) Our new findings indicate that osmotically induced ΔN-TRPV1 channel translocation to the plasma membrane may be a crucial part of the MNC adaptation to sustained increases in osmolality. Osmotically induced ΔN-TRPV1 translocation, like hypertrophy, depends on the activation of PLC and PKC and on exocytotic membrane fusion. Translocation is reversible, and ΔN-TRPV1 membrane staining returns to near-normal levels following the return to isotonic solution for 30 min. Isotonic recovery leads to a dramatic increase in the intracellular staining for ΔN-TRPV1 due to internalization of channels that were labeled on the MNC cell surface. As with MNC hypertrophy, channel internalization is blocked by dynasore, indicating that it involves an endocytic process. Results using patch clamp recordings of ΔN-TRPV1-mediated currents are consistent with those expected for osmotically induced translocation of ΔN-TRPV1 channels to the MNC plasma membrane. Exposure of isolated MNCs to increases in osmolality for 10 min causes an increase in ΔN-TRPV1 currents, presumably due to mechanical activation of the channels^5^^,^^6^ and the potentiation caused by the activation of PLCδ1,^27^ but exposure to increases in osmolality for 90 min causes a significantly greater increase in current, suggesting that another slower process contributes to the increase. That increase, like hypertrophy and ΔN-TRPV1 translocation to the MNC plasma membrane, is largely reversed by 30 min in isotonic solution. There is no significant increase in ΔN-TRPV1 currents in the presence of the PLC inhibitor U73122, which suggests that the processes contributing to both the faster and slower increases in current are dependent on PLC. The increase in current caused by a 90-min exposure to hypertonic solution is significantly lower when exocytotic fusion is blocked, which is consistent with a role for ΔN-TRPV1 channel translocation in the increase. The electrophysiological data therefore supports our hypothesis that prolonged exposure to hypertonic solution evokes ΔN-TRPV1 translocation to the MNC plasma membrane and that this contributes to the increase in ΔN-TRPV1-mediated current that occurs during sustained increases in osmolality. Our experiments were performed at room temperature, and it is possible that ΔN-TRPV1 channel translocation and retrieval could occur more rapidly *in vivo*.

Experiments with MNCs isolated from water-deprived rats and mice demonstrate that this phenomenon is not limited to acutely isolated MNCs treated with hypertonic solutions *in vitro*. MNCs that were isolated from rats and mice deprived of water for 24 h (and that were maintained in hypertonic saline throughout the isolation procedure to prevent recovery) were much larger in cross-sectional area than the MNCs that were exposed to 90 min of hypertonic solution. When returned to isotonic solution, these MNCs showed a rapid decrease in CSA, a decrease in plasma membrane immunoreactivity for ΔN-TRPV1, and an increase in internal ΔN-TRPV1 staining. These results indicate that similar mechanisms are involved in hypertrophy in isolated MNCs and in MNCs *in vivo* even though the increase in CSA observed in isolated MNCs is much smaller (around 5–10%; see Figure 1 and Shah et al.^12^) than that observed *in vivo* following 24 h of water deprivation (see Figures 5 and 6). Electron micrographic studies show that the CSA of MNCs can be increased by water deprivation of only 2 h and that this increase progresses to a near doubling of CSA following a week of water deprivation.^33^ The injection of hypertonic saline into rats can cause some structural changes in as little as 30 min,^34^ which suggests that the time course of the osmotically induced changes that we observe in isolated MNCs is relevant *in vivo*.

MNCs isolated from water-deprived rats showed greater ΔN-TRPV1 current and a greater total cell capacitance than did MNCs isolated from normally hydrated MNCs that were exposed to hypertonic solution for 90 min. These observations are consistent with the imaging experiments showing MNC hypertrophy and an increase in ΔN-TRPV1 labeling in the MNC plasma membrane (Figures 1 and 2) and demonstrate that ΔN-TRPV1 channels remain active despite the increase in cell size. MNC hypertrophy and ΔN-TRPV1 translocation to the MNC plasma membrane therefore occur *in vivo* during water deprivation and to greater extents than what is observed in isolated MNCs following 90-min incubation in hypertonic saline.

We demonstrate that both MNC hypertrophy and the trafficking of ΔN-TRPV1 channels to the plasma membrane are rapidly reversed following treatment with the Na^+^ channel blocker TTX or the L-type Ca^2+^ channel blocker nifedipine. This is consistent with our earlier finding that hypertrophy is reversed by TTX or nifedipine^12^ and suggests that both hypertrophy and the increase in ΔN-TRPV1 channels in the plasma membrane depend on continued action potential firing and influx of Ca^2+^ through L-type Ca^2+^ channels. The implication that the activation of PLC may depend on Ca^2+^ influx is consistent with our previous findings relating to osmotically evoked MNC hypertrophy.^12^ The mechanism by which PLC activity maintains the MNCs in the osmotically activated state is not known.

The Ca^2+^ dependence of these processes is consistent with our observation that neither hypertrophy nor ΔN-TRPV1 translocation occurs in MNCs isolated from transgenic mice that do not express PLCδ1, which is a Ca^2+^-dependent subtype of PLC.^35^ We showed previously that PLCδ1 KO mice show a reduced activation of ΔN-TRPV1 currents in response to increases in osmolality and lack the enhancement of F-actin observed in control mice following treatment with hypertonic solution or angiotensin II.^27^ This enhancement may be important in regulating the sensitivity of the ΔN-TRPV1 channels to changes in mechanical force.^36^^,^^37^ We demonstrate here that MNCs isolated from PLCδ1 KO mice also lack the osmotically induced translocation of ΔN-TRPV1 channels to the MNC plasma membrane. These data suggest that PLCδ1 may be a key trigger for multiple processes involved in MNC osmosensation and osmotransduction. PLCδ1 is known to be involved in a variety of physiological functions in different cell types (including in sperm, skin, and cardiac myocytes), but prior to our recent observations in MNCs,^27^ little was known about the role of PLCδ1 in neurons.^38^^,^^39^ The importance of PLCδ1 in triggering channel translocation in MNCs raises the possibility that PLCδ1 may regulate the trafficking of TRPV1 channels and other TRP channels that occurs in other neurons.^40^ PKC has been shown to activate TRPV1 translocation in dorsal root ganglion cells,^22^ for example, and PLCδ1 could lead to the activation of PKC in these or other cells.

The mechanical activation of ΔN-TRPV1 channels in MNCs has been proposed to rely on a “push-pull” mechanism that depends on a physical interaction between the channels and tubulin molecules in the cytoskeleton.^8^^,^^41^ Osmotically induced cell shrinkage brings the MNC plasma membrane, and therefore the channels, closer to the cytoskeleton, which generates a force on the channel leading to channel opening. It is not clear how this interaction is altered by MNC hypertrophy. Although the increase in total area of plasma membrane might be expected to decrease the mechanical forces exerted on the ΔN-TRPV1 channels through their interactions with tubulin molecules in the cytoskeleton, hypertrophy requires a complex re-adjustment of cytoplasmic volume and ionic composition and could involve concomitant enlargement of the cytoskeleton, which could counteract the changes in mechanical forces on the channels. The MNC cytoskeleton and the relationship between the cytoskeleton and ΔN-TRPV1 channels have been shown to be enhanced by the PLC pathway.^36^^,^^37^ We have shown that PLC is activated in MNCs by increases in osmolality^12^^,^^25^ and that the osmotically induced enhancement of the actin cytoskeleton is defective in PLCδ1 KO mice.^27^ This enhancement might compensate for any decreases in mechanical forces exerted on the channels following MNC hypertrophy. ΔN-TRPV1 channels have been shown to be more sensitive to mechanical stimulation following salt loading in rats.^42^ Whatever the underlying mechanisms, our data demonstrate that MNCs in the hypertrophied state remain sensitive to decreases in osmolality since they undergo rapid recovery when exposed to isotonic solution. As noted above, the maintenance of the MNCs in the osmotically activated state depends on the continued firing of action potentials, influx of Ca^2+^, and activation of PLC. This suggests that a return to isotonic solution deactivates the ΔN-TRPV1 channels leading to decreased action potential firing, decreased Ca^2+^ influx, decreased PLC activity, recovery from hypertrophy, and ΔN-TRPV1 channel internalization.

Osmotically induced PLC activation could contribute to an increase in MNC electrical activity during sustained increases in osmolality in multiple ways, including by altering the cytoskeleton and how it interacts with ΔN-TRPV1 channels, by activating the channels directly, and by inducing the translocation of the channels to the MNC plasma membrane. PLCδ1 KO mice show a markedly greater increase in plasma osmolality following 24 h of water deprivation compared to control mice,^27^ and this may result from any or all of these mechanisms. Further studies will be required to determine the relative importance of these mechanisms in osmoregulation during sustained osmotic challenges and the role of PLCδ1 in those mechanisms.

Osmoregulation is an essential homeostatic mechanism. Dysregulation of osmotic balance leading to chronic increases in osmolality is among the most encountered problems in clinical medicine and is of particular concern in the elderly, the chronically ill, and patients undergoing certain types of drug treatments.^43^ It is therefore important for us to understand the mechanisms underlying the adaptation that MNCs undergo in response to sustained increases in osmolality. Our data suggest that the translocation of ΔN-TRPV1 channels to the MNC cell surface may make an important contribution to the enhanced excitability of MNCs during sustained increases in plasma osmolality and that the enzyme PLCδ1 may play a critical role in triggering that translocation.

### Limitations of the study

We have demonstrated that sustained increases in external osmolality cause translocation of ΔN-TRPV1 channels from internal stores to the plasma membrane of MNCs. This occurs following exposure of isolated MNCs to hypertonic solution and *in vivo* in response to water deprivation of rats or mice. We confirmed using electrophysiological techniques that MNCs treated in these ways show greater current amplitude and infer that the cells should be more excitable. We have not, however, confirmed that the translocation of channels to the MNC plasma membrane contributes to an increase in MNC firing *in vivo* or an increase in VP release during sustained increases in osmolality. Confirmation of this contribution will require the development of tools to specifically block translocation of channels *in vivo* without altering ΔN-TRPV1 channel function.

## STAR★Methods

### Key resources table

**Table:** 

REAGENT or RESOURCE	SOURCE	IDENTIFIER
**Antibodies**		

Anti-Rat TRPV1 (VR1) (extracellular) Antibody	Alomone Labs	Cat# ACC-029, RRID:AB_2040258
Anti-Rabbit IgG (H+L), highly cross-adsorbed, CF™488A antibody produced in donkey	Sigma-Aldrich	Cat# SAB4600036, RRID:AB_2728116

**Chemicals, peptides, and recombinant proteins**		

Bisindolylmaleimide I	Sigma-Aldrich	Cat# 203290, CAS ID: 133052-90-1
Dynasore	Sigma-Aldrich	Cat# D7693, CAS ID: 1202867-00-2
Exo-1	Cayman Chemicals	CAS ID: 75541-83-2
Na_2_-ATP	Sigma-Aldrich	Cat# A26209, CAS ID: 34369-07-8
Na-GTP	Sigma-Aldrich	Cat# 51120, CAS ID: 36051-31-7
Nifedipine	Sigma-Aldrich	Cat# N7634, CAS ID: 21829-25-4
TAT-NSF700	AnaSpec	Cat# AS62238
Tetrodotoxin	Sigma-Aldrich	Cat# 554412, CAS ID: 18660-81-6
Trypsin from Porcine Pancreas	Sigma-Aldrich	Cat# T4799, CAS ID: 9002-07-7
U-73122	Sigma-Aldrich	Cat# U6756, CAS ID: 112648-68-7
U-73343	Sigma-Aldrich	Cat# U6881, CAS ID: 142878-12-4

**Experimental models: Organisms/strains**		

Rat: Long Evans: Crl:LE	Charles River Laboratories	RRID:RGD_2308852
Mouse: C57BL/6J: C57BL/6NCrl	Charles River Laboratories	RRID:IMSR_CRL:027
Mouse: PLCδ1 knockout (KO): Plcd1^tm1Tta^/Plcd1^tm1Tta^	Kiyoko Fukami, Tokyo University of Pharmacy and Life Sciences, Tokyo, Japan	RRID:MGI:2670617

**Software and algorithms**		

GraphPad Prism v6.01	GraphPad Software, LLC	RRID: SCR_002798, https://www.graphpad.com
ImageJ	NIH	RRID:SCR_003070, https://imagej.nih.gov/ij/
Microsoft Excel	Microsoft	RRID:SCR_016137
Biorender	Biorender.com	RRID:SCR_018361
Origin v8.1	OriginLab®	RRID:SCR_002815
Clampfit v10.0 (pClamp)	Axon Instruments®	RRID:SCR_011323

### Resource availability

#### Lead contact

Further information and any requests for reagents may be directed to and fulfilled by the corresponding author, Dr. Thomas E. Fisher (thomas.fisher@usask.ca). Use of the PLCδ1 knockout mouse will require a Material Transfer Agreement from the Laboratory of Genome and Biosignals, School of Life Sciences, Tokyo University of Pharmacy and Life Sciences.

#### Materials availability

This study did not generate new or unique reagents.

### Experimental model and subject details

All procedures that involved animals adhered to the Canadian Council on Animal Care guidelines for humane animal use and were approved by the University of Saskatchewan's Animal Research Ethics Board (AUP # 20010066). Male Long Evans rats and C57BL/6J mice were purchased from the Charles River Laboratories (Laval, QC, Canada) at 6-8 weeks of age. Homozygous PLCδ1 knockout (KO) mice^26^ were bred at 6-8 weeks of age. Upon delivery to our animal facility, mice were individually housed under identical conditions with a standard 24-hour light-dark cycle for 2-4 weeks. The experiments were performed from mice at 8-14 weeks of age. Water deprived animals had water access removed from their cages 24 hours prior to experimentation. Food was provided *ad libitum*, and water was provided *ad libitum* for the normally hydrated animals. Animals were anaesthetized using isoflurane.

### Method details

#### Isolation of MNCs

MNCs were isolated as previously described.^25^ Neurons with a maximal cross-sectional area greater than 160 μm^2^ were identified as MNCs, which is a criterion that is valid for rat^4^ and mouse MNCs.^30^ We did not differentiate between vasopressin and oxytocin expressing MNCs, as both types have been shown to be sensitive to changes in osmolality.^44^ Isolation of MNCs using a similar method resulted in isolated MNCs that were mostly (approximately 80%) vasopressin expressing.^30^

#### Solutions

The isotonic extracellular solution contained (in mM): 135 NaCl, 5 KCl, 1 MgCl_2_, 2 CaCl_2_, 10 glucose, 10 HEPES, pH = 7.4 by NaOH. The intracellular solution contained (in mM): 125 KCl, 10 HEPES, 1 MgCl_2_, 4 Na_2_-ATP, 1 Na-GTP, 14 Tris-Phosphocreatine, 0.5 EGTA, pH adjusted to 7.2 by KOH. The osmolalities of solutions were adjusted with mannitol to 295 (external for rat) or 310 (external for mouse) and 280 mosmol kg^−1^ (internal), respectively. The hypertonic external solution was adjusted to 325 (rat) or 340 (mouse) mosmol kg^−1^ by the addition of mannitol.

#### Chemicals and reagents

All chemicals and reagents were purchased from Sigma-Aldrich (Oakville, ON, Canada), except as noted. Reagents were dissolved in distilled water or DMSO and diluted to their final concentrations in bath solution. U73122 (662035) and Bisindolylmaleimide I (203290) were both were used at a final concentration of 1 μM.^12^ TTX was used at a final concentration of 0.2 μM. Nifedipine was used at a final concentration of 10 μM. Exo-1 (18455) was purchased from Cayman Chemical (Ann Arbor, Michigan 48108 USA) and used at a final concentration of 80 μM.^28^ TAT-NSF700 (3418-0100) was purchased from Innoprep (San Diego, CA 92121, USA) and was used at a final concentration of 1.2 μM.^12^^,^^29^ All four drugs were applied during the entire 90-minute exposure to hypertonic solution in the experiments described in Figures 1 and 2 and for 90 minutes during the experiments in isotonic solution.

#### TRPV1 immunocytochemistry

We used an antibody directed against an external epitope on the TRPV1 channel (Alomone Laboratories, Jerusalem, Israel; catalogue number ACC-029), which enabled us to selectively label channels on the cell surface of live MNCs. This antibody has been used widely, including in experiments to identify TRPV1 in large dense-core granules and on the cell surface of trigeminal ganglion neurons,^21^ on the surface of neurons of the nucleus tractus solitarii,^45^ and on the surface of dorsal root ganglion neurons.^46^ This approach also enabled experiments in which cells were treated with hypertonic solution and then isotonic solution to test whether such recovery involves the internalization of channels. We validated the antibody specificity by incubating isolated MNCs in the antibody with and without the antigen used to generate the antibodies. Inclusion of the antigen decreased the plasma membrane labeling by almost 80% (from 20.4 ± 1.6 to 4.5 ± 0.7 a.u.; n = 5). We also showed that labeling of a cell type that does not express TRPV1 (Hek293 cells) is much lower than that observed in MNCs (20.4 +/- 1.6 vs 3.8 +/- 0.8 a. u.; n = 5). These data demonstrate that the non-specific binding of the antibody is low. MNCs were acutely isolated from rats, control mice, and PLC δ 1 KO mice were pipetted into labeled dishes and allowed to settle for 20 minutes at room temperature. Dishes were treated with 200 μ L of either isotonic (295 mOsm kg⁻^1^ for rats, 310 mOsm kg ^-1^ for mice) or hypertonic (325 mOsm kg⁻^1^ for rats, 340 mOsm kg^-1^ for mice) oxygenated Pipes and incubated for 15 minutes at room temperature. Treatment solution was removed, and either isotonic or hypertonic blocking solution (oxygenated Pipes + 5% donkey serum) was added and incubated for 30 minutes. Next, blocking solution was removed and either isotonic or hypertonic primary antibody solution (oxygenated Pipes + 5% donkey serum + 3.3 μ M anti-rat TRPV1 external epitope antibody) was added and incubated for 15 minutes. Antibody solution was removed, and dishes were washed 3 times with either isotonic or hypertonic oxygenated phosphate-buffered solution (PBS). The PBS was removed, and cells were fixated for 20 minutes using 4% paraformaldehyde (PFA). The PFA was removed, and plates were washed 3 times with PBS + 0.1% Triton X-100 (PBST) to permeabilize the cells. The PBST was removed, secondary antibody solution (PGC + 5% donkey serum + 2 μ M donkey anti-rabbit 488 nm light-sensitive secondary antibody from Sigma-Aldrich in St. Louis, Missouri, USA) was added, and the dishes were incubated in complete darkness for 1 hour. After the secondary antibody solution was removed, plates were washed 3 times with PBST and once with PBS. The PBS was removed and Citifluor AF-1 antifade mounting solution (from Citifluor in Hatfield, Pennsylvania, USA) was added. Isolated rat and mouse MNCs were treated with hypertonic solution for 90 minutes to cause hypertrophy and with isotonic solution for 30 minutes to cause recovery, as these treatments have been shown to cause significant changes.^12^ Images were obtained using a Zeiss LSM700 laser scanning confocal microscope (Carl Zeiss) and Z-stack images were collected for cells deemed to be healthy MNCs.

#### Patch clamp recording

Whole-cell patch clamp recordings were performed at room temperature 2-4 hours after cell isolation. Glass microelectrodes (1.5 x 0.86mm, Sutter Borosilicate Glass, Novato, CA) were pulled with a P-97 horizontal pipette puller (Sutter Instrument Company; Novato, CA). The pipette resistance was between 2.5 and 3.5 MΩ. Signals were amplified by using an EPC-9 amplifier (HEKA Elektronik; Lambrecht/Pfalz, Germany). Using the amplifier circuitry series resistance was compensated 75-80%. In voltage-clamp experiments, ramp voltage commands (-100 to -20 mV, 5 sec, V_h_= -60 mV, 1 kHz sampling) were applied to the cells. Junction potentials were corrected on recording.

### Quantification and statistical analyses

#### Analysis of immunocytochemistry images

All captured images were analyzed using ImageJ software (NIH). Plasma membrane TRPV1 immunofluorescence and cross-sectional area were measured by tracing the perimeter of each MNC by following the line of greatest fluorescence and determining the mean fluorescence of pixels on that line. Intracellular TRPV1 immunofluorescence was measured by tracing the inside of the perimeter of each MNC and determining the mean fluorescence of the pixels within the trace. Data was stored in Microsoft Excel. Graphical and statistical analyses of the immunocytochemical experiments were completed using Origin 8.1.

#### Analysis of electrophysiological recording

Recordings were analyzed using Clampfit 10.0 (Axon Instruments Inc), GraphPad Prism 6.01 (La Jolla, CA) and Origin 8.1 (OriginLab, Northampton, MA).

#### Analysis of all data

All data were expressed as means ± SD. A significant difference was determined by Student’s t-test, or one-way ANOVA with Bonferroni *post hoc* test, as appropriate. N and n indicate the number of animals and cells, respectively. p < 0.05 was considered statistically significant. The level of significance is indicated in graphs using asterisks – ∗ indicates p < 0.05, ∗∗ indicate p < 0.01, ∗∗∗ indicate p < 0.001, and the absence of an asterisk indicates no significant difference.

## Data Availability

• All data reported in this paper will be shared by the lead contact upon request. • This paper does not report original code. • Any additional information required to reanalyze the data reported in this paper is available from the lead contact upon request.

## Supplemental information

Table S1. Summary of figure data, related to Figures 1–7
